# Editorial: How can neuroscience help to turn the tide of the climate crisis?

**DOI:** 10.3389/fnbeh.2023.1193106

**Published:** 2023-04-12

**Authors:** Kinga Igloi, Karim N'Diaye, Eric Burguiere

**Affiliations:** ^1^Basic Neurosciences Department, University of Geneva, Geneva, Switzerland; ^2^Sorbonne Université, Institut du Cerveau—Paris Brain Institute—ICM, Inserm, CNRS, AP-HP, Hôpital de la Pitié Salpêtrière, Paris, France

**Keywords:** climate, cognitive bias, neurophysiological adaptation, behavioral neuroscience, decision making

The climate crisis is one of the most pressing challenges of our time, and it needs to be addressed from different perspectives. How does it come that the human brain cannot interpret this menace and act against it quickly to avoid the (massive) harm that may ensue? Fundamentally it is up to each individual on our planet, from farmers, to city residents and decision makers that could add up to ensure global behavioral changes toward more sustainable environmental policies. The scope of this topic is to bring together researchers from different neuroscientific viewpoints and integrative levels (from molecular neuroscience to human behavior), with the aim to find new approaches that may help act on the climate crisis from a neuroscientific perspective. The four published articles take different angles to address this issue, two articles focus on adaptive strategies: one showing organisms modification in fruitflies for phototactic choices depending on environmental lighting conditions (Krams et al.) and the other at a more general cellular, molecular and functional levels of marine species adaptation in response to ocean acidification (Michaiel and Bernard). These articles illustrate existing mechanisms that may mediate adaptive responses to environmental changes. The two other articles focus on how neuroscience may explain and possibly influence our environmental-related choices and behaviors. Leeuwis et al. address neuroscientific underpinnings of consumer attitude and how this attitude could be acted on using affective conditioning techniques. Munuera and Burguière focus on the dopaminergic nervous system and how it is involved in cognitive biases that partially mediate our inconsistent behaviors regarding climate change, and explains why it difficult for us to act now for something that will happen in a long time. These articles shed light on how neuroscience may explain our current behaviors, but importantly also suggest new avenues based on neuroscientific methods that may help modifying our behaviors (by strategic changes using conditioning techniques, as in Leeuwis et al.) but also through the interaction of the dopaminergic system with higher-order cognitive functions that may influence positively our social behaviors if linked to short-time pro-environmental outcomes (Munuera and Burguière).

By exploring the neuroscientific backgrounds of adaptive behaviors and of complex decision-making processes that relate to sustainable and less-sustainable decisions in our everyday lives, neuroscience (including behavioral science), is a crucial and so-far little-recognized component that could help turn around the tide of the climate crisis. Cognitive biases and nudging techniques that are at the core of behavioral approaches to consumer-related behavior in the climate crisis would largely benefit from being promoted further to the general public. Neuroscience can provide insights into the cognitive and emotional processes that underlie pro and anti-sustainable behaviors but also climate change denial. Taken together, the selection of these articles provides an illustration of the current neuroscientific potential of adaptive and behavioral components related to the fight against climate change.

## Author contributions

All authors listed have made a substantial, direct, and intellectual contribution to the work and approved it for publication.

